# Post-Traumatic Stress Disorder in Fort McMurray: Prevalence and Correlates One Year After the Flood

**DOI:** 10.1192/j.eurpsy.2023.1021

**Published:** 2023-07-19

**Authors:** W. Mao, E. Eboreime, R. Shalaby, N. Nkire, B. Agyapong, H. Pazderka, G. Obuobi-Donkor, M. Adu, E. Owusu, F. Oluwasina, Y. Zhang, V. Agyapong

**Affiliations:** 1 Psychiatry, University of Alberta, Edmonton; 2 Psychiatry, Dalhousie University, Halifax, Canada

## Abstract

**Introduction:**

As a result of the floods in Fort McMurray (FMM) and the surrounding area in 2020, more than $228 million in insured damage was sustained, over 1200 structures were damaged, and more than 13,000 people were evacuated from their homes.

**Objectives:**

The aim of the study was to determine the prevalence of post-traumatic stress disorder (PTSD)-like symptoms among the population of FMM one year after the flood in 2020 and the risk predictors associated with such symptoms.

**Methods:**

In order to collect sociodemographic, clinical, and flood-related information from residents of FMM via REDCap, an online quantitative cross-sectional survey was administered between April 24 and June 02, 2021. Respondents were assessed for probable PTSD using the PTSD Checklist for DSM-5 (PCL-C).

**Results:**

An overall response rate of 74.7% was achieved among 186 of 249 respondents. The prevalence of likely post-traumatic stress disorder was 39.6% (65). There was a significantly increased likelihood of developing PTSD symptoms in respondents with a history of depression (OR= 5.71; 95% CI: 1.68 - 19.36). As well, disaster responders experiencing limited and no family support after the disaster were more likely to report PTSD symptoms (OR= 2.87; 95% CI: 1.02 - 8.05) and (OR= 2.87; 95% CI: 1.06 - 7.74), respectively.

**Conclusions:**

As a result of our study, we found that those who had a history of depression and had sought health counseling were significantly more likely to develop PTSD symptoms following flooding, while those with family support were less likely to suffer from PTSD symptoms. There is a need for further studies to investigate the relationship between the need for counseling and the presentation of potential symptoms of post-traumatic stress disorder.

**Disclosure of Interest:**

None Declared

